# Assessment and Prediction of Salivary Gland Function After Head and Neck Radiotherapy: A Systematic Review

**DOI:** 10.1002/cam4.70494

**Published:** 2024-12-16

**Authors:** J. Le Guevelou, X. Palard‐Novello, E. Kammerer, M. Baty, M. Perazzi, A. Larnaudie, R. De Crevoisier, J. Castelli

**Affiliations:** ^1^ Department of Radiotherapy Centre Eugène Marquis Rennes France; ^2^ Department of Nuclear Medicine Centre Eugène Marquis Rennes France; ^3^ Department of Radiotherapy Centre François Baclesse Caen France

**Keywords:** head and neck cancer, MRI, PET/CT, radiotherapy, salivary gland, xerostomia

## Abstract

**Background:**

Modern imaging techniques with magnetic resonance imaging (MRI) or positron emission tomography/computed tomography (PET/CT) have recently been developed to assess radiation‐induced damage to salivary structures. The primary aim of this review was to summarize evidence on the imaging modalities used for the assessment and prediction of xerostomia after head and neck radiotherapy (RT).

**Methods:**

A systematic review of the literature was performed using successively the MeSH terms “PET,” “MRI,” “scintigraphy,” “xerostomia,” and “radiotherapy.”

**Results:**

Salivary excretion flow following head and neck RT is correlated with the dose delivered to both parotid and submandibular glands. Salivary gland standardized uptake value extracted from PET/CT following RT has been shown to be correlated with SEF. Models including early SUV decline or ADC increase during RT and clinical parameters can help predict the loss of salivary function after RT.

**Conclusions:**

Modern imaging parameters appear to be correlated with salivary gland scintigraphy parameters. Models including functional parameters extracted from either PET/CT or MRI unveil new possibilities for adaptive treatment in a selected population of patients.

## 1 Introduction

Xerostomia represents one of the most frequent late sequelae after head and neck radiotherapy (RT), due to the high radiosensitivity of salivary glands [1]. Despite modern RT techniques with both intensity‐modulated radiotherapy (IMRT) and image‐guided radiotherapy (IGRT), up to 40% of patients will experience moderate to severe late xerostomia, negatively impacting the quality of life [2, 3]. Several studies have investigated dose‐volume parameters correlated with salivary gland damage. To date, the consensus for parotid gland‐sparing is to achieve a mean dose < 26–30 Gy [4]. However, doses as low as 10–15 Gy can result in late severe xerostomia [5], suggesting either the role of individual sensitivity factors or radiosensitivity heterogeneity within the organ. Moreover, the existence of a dose threshold for submandibular gland sparing is still lacking.

Many studies have tried to elucidate the mechanism of radiation‐induced lesions in the salivary glands. Edematous stenosis of the major salivary duct has long been suggested to be a leading cause of xerostomia occurring during RT [6]. Damage to both acinar and ductal systems has been shown to be the main cause of late xerostomia [7].

Several reliable methods exist when it comes to quantification of salivary gland function. Salivary flow rate measurement represents of one the most employed methods [8]. Other approaches include the use of salivary gland scintigraphy (SGS) to measure salivary excretion fraction (SEF) [9]. These quantitative techniques however remain hampered by a weak correlation with patient‐reported outcomes [10] and fail to provide spatial information on salivary dysfunction and damage localization. More modern imaging techniques (positron emission tomography/computed tomography (PET/CT), magnetic resonance imaging (MRI)) have been developed to assess radiation‐induced damage to salivary structures.

The primary aim of this review was to summarize evidence on the imaging modalities used for the assessment and prediction of xerostomia after head and neck radiotherapy.

## 2 Material and Methods

### 2.1 Eligibility Criteria

Studies reporting the correlation between imaging modalities and the onset of xerostomia after head and neck radiotherapy were deemed eligible. Articles whose primary aim was to assess a treatment modality (3D‐CRT vs. IMRT, unilateral prophylactic neck irradiation…) were excluded.

### 2.2 Search Strategy

A systematic review of the literature was performed on October 2024 on the PubMed database, using successively the MeSH terms “PET,” “MRI,” “scintigraphy,” “xerostomia,” and “radiotherapy.” Limitations were placed on articles published before 2000, to gather homogeneous data. For every study, the following data were retrieved: date of publication, number of patients included, study design, radiation dose and technique, median follow‐up, and toxicity outcomes.

### 2.3 Synthesis Method

This systematic review was performed in accordance with the Preferred Reporting Items for Systematic Reviews and Meta‐Analyses guidelines [11].

## 3 Results

### 3.1 Study Selection

**FIGURE 1:**
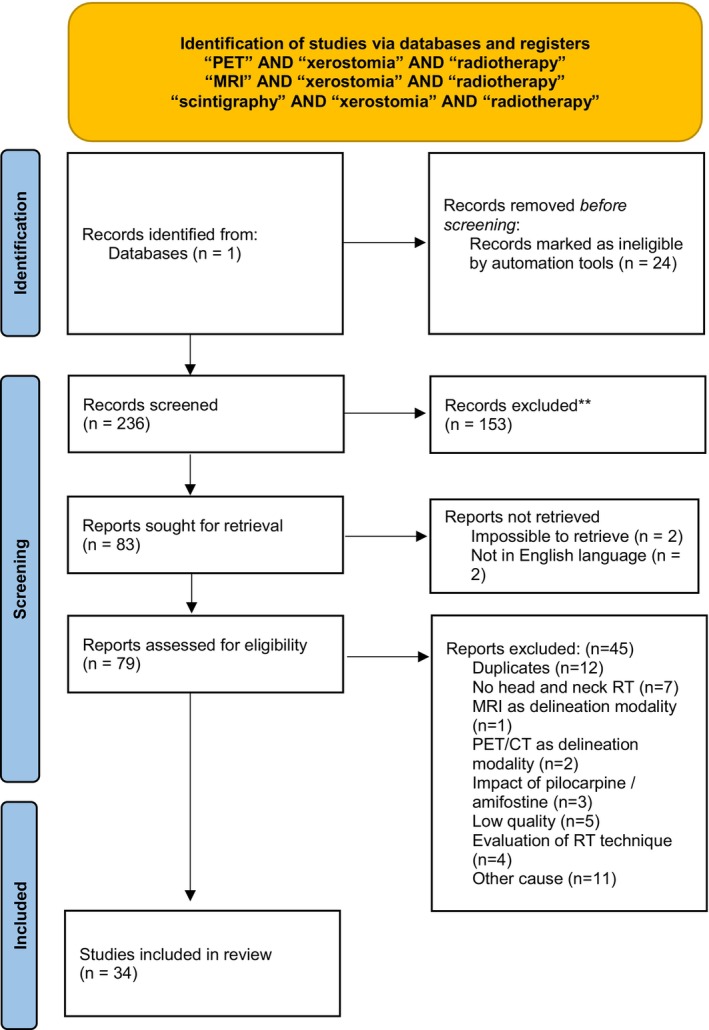
Flowchart of the study.

A flowchart of the literature screening is shown in Figure 1. The search allowed us to retrieve a total of 236 articles. After screening, 153 records that did not address the evaluation of xerostomia after head and neck RT were excluded (case reports, guidelines, xerostomia treatment, reviews, etc.), leaving a total of 83 articles sought for retrieval. Articles not in English or impossible to completely retrieve were excluded, leaving 79 reports assessed for eligibility. A total of 12 duplicates were found. After full‐text reading, 33 additional reports were excluded (assessment of imaging for delineation purposes, low quality, low number of patients, etc.). Finally, 34 articles were included in the present review.

### 3.2 Salivary Gland Scintigraphy

SGS represents one of the gold standard approaches to assess salivary function following head and neck RT. A total of 7 studies implemented this imaging modality to investigate the tolerance dose of salivary glands, after head and neck RT (Table 1). These studies observed a correlation between mean doses delivered to salivary glands and the reduction in salivary excretion rate.

**TABLE 1:** Studies assessing salivary glands scintigraphy for the investigation of xerostomia following head and neck RT.

Author	Design	Number of patients	RT dose	RT technique	Imaging modality	Median follow‐up	Main result
Chen et al. [12]	Prospective study	31 pts. Nasopharynx/oropharynx/larynx/parotid/oral cavity	Mean dose ipsilateral PG: 51.7 Gy Mean dose contralateral PG: 36.7 Gy	IMRT	Salivary gland scintigraphy Baseline/1 year/2 years after RT	24 months	Correlation between mean PG dose and reduction of salivary excretion function at 1 year/2 years PG TD50 assessed at 1 year after RT: 43.6Gy Contralateral PG and SG preservation: reduction in rates of sticky saliva and improved quality of life (EORTC QLQ HN35)
Murthy et al. [13]	Prospective study	90 pts	Mean dose ipsilateral SG: 61 Gy Mean dose contralateral SG: 51 Gy	IMRT	Salivary gland scintigraphy Baseline/every 6 months after RT	24 months	Nadir in salivary excretion fraction at 12 months (15%). Progressive recovery up to 38% at 24 months. Inverse correlation between salivary excretion fraction ratio and mean SG dose at all timepoints. TD50 at 1 year: 36 Gy, TD50 at 2 years: 44 Gy
Lee et al. [14]	Prospective study	31 pts. Nasopharynx/oropharynx/larynx/parotid	Mean dose ipsilateral PG: 51.7 Gy Mean dose contralateral PG: 36.7 Gy	IMRT	Salivary gland scintigraphy Baseline/3 months/1 year after RT	12 months	Mean dose delivered to PG correlated with recovery of salivary function at 1 year. PG TD50: 43.6Gy
Lee et al. [15]	Prospective study	35 pts. Oropharynx /oral cavity/nasopharynx/nasal cavity	Mean dose PG: 23.1Gy Mean dose SG: 54.6Gy	IMRT	Salivary gland scintigraphy Baseline/6 weeks/3 months after RT	3 months	Mean change in PG excretion rate: −55% and −28% at 6 weeks and 3 months, respectively Mean change in SG excretion rate: −76% and −85% at 6 weeks and 3 months, respectively Mean dose PG ≥ 23 Gy correlated with a > 50% decrease in salivary excretion rate at 6 weeks and 3 months. Mean dose PG ≥ 30 Gy correlated with a > 50% decrease in salivary excretion rate at 3 months. Mean dose PG ≥ 23 Gy correlated with a > 75% decrease in salivary excretion rate at 6 weeks. Mean dose PG ≥ 30 Gy correlated with a > 75% decrease in salivary excretion rate at 6 weeks and 3 months. Mean dose SG ≥ 30 Gy correlated with a > 75% decrease in salivary excretion rate at 6 weeks Mean dose SG ≥ 30 Gy, 39 Gy and 42 Gy correlated with a > 50% and > 75% decrease in salivary excretion rate at 3 months
Münter et al. [16]	Retrospective study	18 pts. Nasopharynx/oropharynx/larynx/sinus	NR	IMRT	Salivary gland scintigraphy Baseline/after RT	23 months	Significant decrease in relative excretion rate if mean dose PG ≥ 26 Gy and 30 Gy Reduction in PG excretion rate > 50% and > 75%: 50% probability if dose > 34.8 Gy and > 40.8 Gy
Itonaga et al. [17]	Retrospective study	31 pts. Oropharynx/nasopharynx/hypopharynx/larynx/oral cavity/CUP	Mean PG dose: 35.8 Gy	IMRT/3D‐CRT	Salivary gland scintigraphy Baseline/3 months/1 year after RT	12 months	Mean dose delivered to salivary glands: predictive of deterioration of salivary gland function Average tolerated dose to salivary glands: 46 Gy
Stock et al. [18]	Retrospective study	46 pts. Oropharynx/oral cavity	NR	IMRT	Salivary gland scintigraphy Baseline/3 months/6 months/9 months/12 months after RT	12 months	Association between the reduction in relative excretion rate at 3 months and PG mean dose/V26 Gy/V40 Gy Partial recovery up to 6 months after RT for contralateral PG Partial recovery between 6 to 12 months after RT for contralateral PG

Abbreviations: 3D‐CRT = tridimensional conformational radiotherapy, CUP = carcinoma of unknown primitive, PG = parotid gland, SG = submandibular gland.

Three prospective studies assessed the relation between the dose delivered to the parotid and salivary excretion rates. Chen et al. confirmed the existence of a linear correlation between the mean parotid dose and the reduction of salivary excretion rate following head and neck radiotherapy [12]. The TD50 (dose for uniform irradiation that results in a 50% probability of a complication) for the parotid gland was evaluated at 43.6 Gy, similar to the study led by Lee et al. [14]. With regard to the quality‐of‐life outcomes, contralateral salivary gland sparing resulted in a minimization of sticky saliva. Lee et al. found several dosimetric parameters as being associated with SEF decrease at 6 weeks and 3 months, but only a mean parotid dose of 23 Gy was correlated with RTOG toxicity grade ≥ 2 [15]. However, this study is hampered by the short follow‐up time, which does not allow for an evaluation of the recovery in salivary gland function, usually occurring 12 months following RT.

A relationship between the dose delivered to salivary glands and a reduction in salivary function was also observed within 3 retrospective studies. Itonaga et al. assessed salivary glands function after head and neck RT using SGS, and mean washout rates of 49%, 14%, and 29% at baseline, 3 months and 1 year following RT. They also observed the presence of a dose–response relationship between the mean dose delivered to salivary glands and the deterioration of salivary function [17]. Stock et al. showed an association between the reduction in relative SEF at 3 months and several parotid gland dose‐volume parameters (mean dose, V26 Gy, V40 Gy) [18]. A partial recovery was demonstrated for the contralateral parotid gland, occurring up to 6 months following RT. However, the percentage of recovery was not described within the study. In the study led by Münter et al. both mean parotid doses ≥ 26 Gy and ≥ 30 Gy were associated with a significant reduction in relative SEF. A mean parotid dose of 40.8 Gy was associated with a 50% probability of seeing a > 75% reduction in parotid SEF [16].

Fewer quantitative data are available specifically for submandibular glands. Murthy et al. showed a large decrease in SEF ratio (percentage of the baseline SEF) up to 12 months, estimated at 15% [13]. Of note, the mean dose delivered to the ipsilateral and contralateral submandibular gland were estimated at 61 Gy and 51 Gy, respectively. A progressive recovery was observed up to 24 months (38% of the baseline SEF value). Severe xerostomia is defined as a SEF ratio ≤ 45%, the TD50 values for submandibular glands were estimated at 1 and 2 years, respectively, at 36 Gy and 44 Gy.

While these studies all conclude that salivary excretion rate is strongly linked with the dose delivered to salivary glands, the most predictive parameter (either mean dose, V ≥ 26 Gy, V ≥ 30 Gy) varies between studies. Additionally, only one study showed a correlation between dosimetric parameters and RTOG toxicity, which may indicate a difference in perception of xerostomia between patients.

### 3.3 Positron Emission Tomography/Computed Tomography (PET/CT)

A total of 7 studies were led to correlate metabolic imaging biomarkers with either salivary function or salivary toxicity (Table 2). These studies demonstrated a correlation between the dose delivered to salivary glands, and standardized uptake values (SUV).

**TABLE 2:** Studies assessing PET/CT for the investigation of xerostomia following head and neck RT.

Author	Design	Number of patients	RT dose	RT technique	Imaging modality	Median follow‐up	Main result
Cannon et al. [19]	Prospective study	98 pts. Oropharynx/larynx/hypopharynx	Average mean dose PG: 25.4 Gy	IMRT	PET/CT FDG Baseline Post RT: 7–9 weeks	24 months	Fractional loss of PG uptake correlated with early parotid toxicity (both salivary output and RTOG xerostomia scores)
Mohan et al. [20]	Prospective study	28 pts. Oral cavity/larynx/hypopharynx/oral cavity	Mean dose PG: 23.7 Gy Mean dose SG: 51.4 Gy	IMRT/VMAT	PET/CT PSMA Baseline/during RT/1 month/6 months after RT	6 months	Every Gy in both PG and SG: decrease in SUV at 6 months by 1.8%, respectively, PSMA loss correlated with xerostomia at 1 month after RT
Trada et al. [21]	Prospective study	56 pts. Nasopharynx/larynx/hypopharynx/palate/tongue	Mean dose ipsilateral PG: 41 Gy Mean dose contralateral PG: 26 Gy	IMRT	PET/CT FDG Baseline Mid‐treatment	6 months	Most predictive model included: the prescription of chemotherapy, dose to contralateral parotid gland, and ipsilateral evolution of SUV median (baseline/mid‐treatment)
Van Dijk et al. [22]	Prospective study	161 pts. Oral cavity/oropharynx/larynx/nasopharynx/hypopharynx	NR	IMRT/VMAT	PET/CT FDG Baseline	12 months	Textural PET IBM and 90th percentile standardized value at baseline predictors of moderate to severe xerostomia at 12 months
Wilkie et al. [23]	Prospective study	47 pts. Oropharynx	Mean ipsilateral PG dose: 43 Gy Mean contralateral PG dose: 25 Gy	IMRT	PET/CT FDG Baseline	12 months	Correlation between 90th percentile of PG SUV, mean PG dose, and late xerostomia
Elhalawani et al. [24]	Retrospective study	108 pts. Oropharynx	Mean dose ipsilateral PG: 35.4 Gy Mean dose contralateral PG: 20 Gy	IMRT	PET/CT FDG Baseline/3–6 months following RT initiation	Up to 6 months	Reduction in median SUV values after RT Correlation between PG volume receiving > 10–15 Gy and reduction in median SUV values after RT Severe xerostomia at 3–6 months correlated with contralateral PG mean dose
Li et al. [25]	Retrospective study	137 pts. Oropharynx/larynx/hypopharynx/oral cavity	NR	IMRT/VMAT	PET/CT FDG Baseline	12 months	Confirmation in a validation cohort (see Van Dijk et al.): mean SUV and 90th percentile standardized value at baseline predictors of moderate to severe xerostomia at 12 months

Abbreviations: FDG = fluoro deoxy glucose, Gy = Gray, IBM = image biomarker, PET/CT = positron emission tomography/computed tomography, PG = parotid glands, PSMA = prostate‐specific membrane antigen, SUV = standardized uptake value, VMAT = volumetric arc therapy.

Cannon et al. investigated the correlations between quantitative measurements of 18F‐fluorodeoxyglucose (FDG) uptake in parotid and xerostomia, in a large prospective cohort of 98 patients receiving head and neck RT [19]. This study showed a strong positive correlation between parotid FDG uptake and sialometry (stimulated and unstimulated salivary samples assessed every minute for 5 min), and also with clinical outcomes. Indeed, fractional loss of parotid FDG uptake at 7–9 weeks correlated with both post‐RT SEF and RTOG xerostomia scores assessed at the same time point. However, no correlation was demonstrated with patient‐reported outcomes (PRO) (XQL score). A relationship between radiation dose delivered to both parotid and submandibular glands and decrease in SUV was also described using prostate‐specific membrane antigen (PSMA) PET/CT. Each increase of 1 Gy in mean doses delivered to the parotid gland and submandibular glands was associated with a decrease in PSMA SUV at 1 and 6 months [20]. Lastly, Elhalawani et al. assessed within a retrospective study the evolution of parotid FDG uptake after RT for oropharyngeal cancer [24]. A correlation was found between volume receiving 10–15 Gy and the reduction in median SUV. While the mean dose delivered to the ipsilateral parotid gland was usually well above dose constraints (mean dose: 35.4 Gy), the onset of severe xerostomia at 3–6 months was correlated with the dose delivered to the contralateral gland (mean dose in the whole cohort: 20 Gy). However, controversial data exists with regards to SUV evaluation after head and neck RT, as a study showed that the average parotid gland SUV mean was significantly higher at 3 months after treatment compared with pretreatment values, due to inflammation within the parotid gland [26].

Four studies were led with the intent to predict xerostomia after head and neck RT, using multiparametric models including functional data from FDG PET/CT, and showed that high parotid gland uptake was associated with a lower risk of developing xerostomia. Van Dijk et al. developed a model using both baseline PET/CT and baseline parameters (presence or not of xerostomia) obtained prospectively from 161 head and neck cancer patients [27]. Both intensity PET biomarkers and textural features biomarkers were extracted. High parotid gland metabolic activity at baseline (90th percentile value of the intensity PET image biomarker) was associated with a lower risk of developing late severe xerostomia. The same findings were also demonstrated by Wilkie et al. showing a correlation between baseline 90th percentile parotid gland SUV, mean parotid dose, and xerostomia assessed 12 months following RT [23]. Li et al. further validated this model on a large cohort of 137 patients, receiving RT for head and neck carcinoma. Forty percent of the patients developed moderate to severe xerostomia at 12 months [25]. Both 90th percentile SUV and mean SUV to the contralateral parotid gland at baseline were confirmed as predictors for late xerostomia, with an area under the curve (AUC) assessed at 0.71 and 0.70, respectively. Trada et al. also developed a model measuring early functional changes within parotid glands during RT and correlated them with the onset of xerostomia [21]. Fifty‐one percent of the patients developed late severe (grade ≥ 2) xerostomia, according to the CTCAE grading scale. Similarly to the study led by Mouminah et al. [26], an increase in parotid SUV was demonstrated during RT treatment. The integration of both baseline and mid‐FDG PET/CT changes within the ipsilateral parotid gland, together with baseline clinical factors (contralateral parotid dose and prescription of chemotherapy) was highly predictive of the onset of severe xerostomia at 6 months after RT.

### 3.4 Magnetic Resonance Imaging (MRI)

A total of 18 studies were led to the impact of head and neck RT on salivary parenchyma and ducts.

MR sialography has been investigated within two prospective but small (< 15 patients) cohorts, for the evaluation of xerostomia following head and neck RT (Table 3). Astreinidou et al. evaluated whether MR sialography could detect changes in the salivary duct system after head and neck RT [28]. The visibility of the salivary ducts was assessed through the use of a categorical scoring system, dividing the ducts into several portions. The score was 1 if the ducts were visible, and 0 if they were not. At 6 weeks, the trajectory of the salivary ducts was only partially visualized, for both submandibular and parotid glands. The visibility score (in‐house definition) improved at 6 months for some patients but only within the parotid glands. Of note, no dilatation was observed within parotid and submandibular ducts, excluding the hypothesis of an obstructive part in acute xerostomia following RT. The average amount of saliva (produced in 10 min) measured before, 6 weeks, and 6 months after RT was 3.4, 0.2, and 0.1 mL, respectively. The impact of the dose delivered to the salivary gland on imaging parameters was not assessed within this study. Ou et al. also performed MR sialography in a cohort of 14 patients receiving head and neck RT for nasopharyngeal carcinoma [29]. The mean dose delivered to parotid and submandibular glands was 38.9 Gy and 59.3 Gy. They also demonstrated an early decrease in visibility score and response to gustatory stimulation at 1 week post‐RT for both parotid and submandibular glands. At 1 year, the visibility scores only improved for parotid glands, but not for the submandibular glands. No response to gustatory stimulation was demonstrated at 1 year for submandibular glands.

**TABLE 3:** studies assessing MRI for the investigation of xerostomia following head and neck RT.

Author	Design	Number of patients	RT dose	RT technique	Imaging modality	Median follow‐up	Main results
Astreinidou et al. [28]	Prospective study	9 pts. Nasopharynx/oropharynx	Mean dose to PG: 35 Gy Mean dose to SG: 62 Gy	IMRT	MR sialography Baseline/6 weeks/6 months after RT	Up to 6 months	Reduced visibility of parotid and salivary glands ducts at 6 months Improvement in visibility score at 6 months for some PG, but not for SG
Dirix et al. [30]	Prospective study	8 pts. Oral cavity/oropharynx/CUP	Mean dose contralateral PG: 20 Gy Mean dose ipsilateral PG: 53.9 Gy Mean dose contralateral SG: 47.5 Gy Mean dose ipsilateral SG: 58.5 Gy	IMRT	DW MRI with gustatory stimulation Baseline/9 months after RT	Up to 9 months	Baseline ADC values significantly higher after RT in ipsilateral PG. Response to gustatory stimulation in contralateral PG Absence of response to gustatory stimulation in ipsilateral PG and SG
Shen et al. [31]	Prospective study	54 pts. Nasopharynx	Mean dose PG: 36.3 Gy	IMRT	Intravoxel incoherent motion (IVIM) MRI Baseline/at the end of RT/3 months after RT	3 months	The D, D* and f values increased significantly from pre‐RT to post‐RT Percentage parotid shrinkage from pre‐RT to post‐RT: 42.67% ± 11.65% The percentage parotid shrinkage mainly occurred in the early stage of RT and was consistent with the changes in IVIM parameters N3 stage (OR 3.553, 95% CI 1.29–9.789) and high pre‐D (OR 23.85, 95% CI 2.392–237.818) were associated with a greater risk of severe xerostomia.
Zhao et al. [49]	Prospective study	97 pts. Nasopharynx	NR	IMRT	DW MRI Baseline/mid‐RT/end of RT/1 month post RT/3 months post RT/6 months post RT/9 months post RT/12 months post RT	12 months	Correlation between the dose delivered to salivary glands and change in ADC ADC lower in parotid and sublingual glands, in patients receiving submandibular glands sparing.
Fan et al. [32]	Prospective study	31 pts. Nasopharynx	Mean dose PG: 29.8–30.6 Gy Mean dose SG: 41.3–46.6 Gy	IMRT	DW MRI Baseline/1 month/3 months/6 months/9 months/12 months after RT	Up to 12 months	ADC post RT higher than ADC pre RT Salivary flow rate negatively correlated to ADC value Higher ADC of patients with severe xerostomia, compared with patients with mild–moderate xerostomia
Juan et al. [33]	Prospective study	19 pts. Nasopharynx/oral cavity/oropharynx/hypopharynx	Mean cumulative dose PG: 36 Gy	IMRT 3D‐CRT	DCE MRI Median interval: 17 months	Variable	Modification of perfusion parameters in irradiated PG (lower Kel and K21, higher values of peak‐enhancement and time to peak)
Juan et al. [34]	Prospective study	11 pts. Nasopharynx	Mean cumulative dose PG: 28 Gy	IMRT	DWI MRI Baseline/median: 1.7 1.7 month/8 months/16 months after RT	Median: 16 months	Reduction in parotid volume. 1.7 months: 31.2%, 8 months: 26.1%, 16 months: 17.1% ADC post RT higher than ADC pre RT Negative correlation between ADC value and parotid volume Positive correlation between ADC value and RT dose
Kan et al. [35]	Prospective study	12 pts. Nasopharynx/larynx/oropharynx/unknown	Median dose PG: 36 Gy	IMRT	Anatomical MRI sequences (T1‐T2) Baseline/during or ≤ 3 weeks after RT	Maximum follow‐up: 3 months	Diminution in maximal cross‐sectional area of the gland, narrowing of the main duct
Loimu et al. [36]	Prospective study	20 pts. Oropharynx/larynx	Mean dose to PG: 30.2–37.4 Gy	IMRT	DWI MRI with gustatory stimulation Baseline/6 months after RT	6 months	Biphasic response to gustatory stimulation, with an initial increase in ADC values and subsequent decrease. Persistence of the biphasic response after RT. Increase in baseline ADC values after RT (vs pre RT).
Marzi et al. [37]	Prospective study	89 pts. (63 analyzed) Oropharynx	Mean dose to SG in pts. with xerostomia < G2: 62.5 Gy Mean dose to SG in pts. with xerostomia G ≥ 2: 64.2 Gy	IMRT	DWI and DCE MRI Baseline/every 6 months for the firsts 2 years after RT	Up to 12 months	Model based on V65 Gy and xerostomia questionnaires predictive of grade 2 xerostomia at 12 months
Ou et al. [29]	Prospective study	14 pts. Nasopharynx	Average mean dose to SG: 59.3 Gy Average mean dose to PG: 38.9 Gy	IMRT	MRI sialography Baseline/1 week/1 year	Up to 12 months	1 week: decreased visibility score of PG (65% baseline) and SG ducts and decreased response to gustatory stimulation 1 year: return to baseline visibility score for the PG ducts (100%)/no visibility of the SG ducts and no response to gustatory stimulation
Shi et al. [38]	Prospective study	30 pts. Nasopharynx	NR	IMRT	DWI MRI with gustatory stimulation Baseline/timeline not reported	Mean follow‐up: 16 months	Baseline and stimulated ADC higher after RT Rest and stimulated salivary flow rate lower after RT Baseline PG ADC after RT correlated with xerostomia scores
Zhang et al. [39]	Prospective study	28 pts. Nasopharynx	Mean dose PG: 40.1 Gy	IMRT	DWI MRI with gustatory stimulation Baseline/2 weeks after RT	NR	Baseline ADC higher after RT Range of response after gustatory stimulation lower after RT
Zhang et al. [40]	Prospective study	26 pts. Nasopharynx	Mean dose PG: 36.4 Gy Mean dose SG: 55 Gy	IMRT	DWI MRI Baseline/at 2 weeks during RT	6 months	Baseline ADC significantly higher after 2 weeks of RT Lower response after gustatory stimulation (lower ADC at stimulation) ADC during gustatory stimulation predictive of xerostomia at 6 months
Zhang et al. [41]	Prospective study	28 pts. Nasopharynx	Mean dose PG: 40.8 Gy Mean dose SG: 59.8 Gy	IMRT	DWI MRI Baseline/1 week after RT	12 months	Increase in PG and SG ADC at 1 week post RT Decrease in PG ADC 1 year after RT Predictor of improvement of xerostomia: dose to PG (OR = 0.6), ADC of SG (OR = 3.3), PG maximal ADC (OR = 0.5), PG time to peak ADC (OR = 0.7)
Zhou et al. [42]	Prospective study	22 pts. Nasopharynx	Mean PG dose: 29.2 Gy	IMRT	DWI MRI Baseline/at 5 weeks during RT/at 4 weeks after RT	4 weeks	Increase in PG ADC values during RT Decrease in PG volume and diffusion kurtosis coefficient values
Zhou et al. [43]	Prospective study	18 pts. Nasopharynx	Mean PG dose: 28.4 Gy	IMRT	Intravoxel incoherent motion MRI Baseline/4 weeks after RT	4 weeks	Increase in all IVIM and DCE MRI parameters after RT Change in perfusion fraction, maximum relative enhancement and ADC negatively correlated with PG atrophy
Van Dijk et al. [27]	Retrospective study	68 pts. Validation on 25 pts. Nasopharynx/oropharynx /hypopharynx/larynx	Average mean dose PG: 22–31.8 Gy	IMRT/VMAT	Anatomical MR sequences Baseline	12 months	Ratio fat/functional PG predictive of xerostomia at 12 months
Wu et al. [44]	Retrospective study	21 pts. Nasopharynx	Mean dose PG: 38 Gy Mean dose SG: 36.9 sGy	IMRT	Ultrasound DCE MRI Baseline/every 6 months up to 2 years after RT	Up to 24 months	Reduction in PG and SG volume (maximal reduction at 6 months) Decrease in resistive index and pulsatility index (maximal reduction at 6 months) Increase in peak systolic velocity and end diastolic velocity (maximal at 6 months)
Sheikh et al. [45]	Retrospective study	266 pts. Oropharynx/oral cavity	Mean PG D40%: 27.8 Gy Mean SG D60%: 56.8 Gy	IMRT	CT/MRI Baseline	3 months	CT, MR and DVH features may help predict the onset of xerostomia

Abbreviations: 3D‐CRT = tridimensional conformational radiotherapy, ADC = apparent diffusion coefficient, CT = computed tomography, CUP = carcinoma of unknown primary, DCE = dynamic contrast‐enhanced, DW = diffusion weighted, G = grade, IMRT = intensity modulated radiotherapy, MR = magnetic resonance, NPC = nasopharyngeal carcinoma, NR = non reported, PG = parotid glands, pts = patients, RT = radiotherapy, SG = submandibular glands, VMAT = volumetric arc therapy.

Other than salivary ducts, modifications in salivary gland parenchyma have been investigated as a way to assess xerostomia following head and neck RT. Early modification in ADC has been shown after only a few weeks after RT. Baseline parameters such as ratio fat/functional parotid gland or baseline ADC have been suggested as predictors of xerostomia after treatment. Salivary gland function assessed with SGS seems to correlate with MRI parameters, such as ADC. The correlation between ADC and dose‐volume parameters is suggested only in one study.

Kan et al. assessed radiation‐induced damage to the parotid gland with high‐resolution MRI performed before and either during or shortly after RT [35]. They showed both an early narrowing of the main parotid duct and a reduction in the size of the gland, with a diminution of the cross‐sectional area. Unfortunately, this study did not include a correlation with dosimetric data or patient‐reported outcomes.

Diffusion‐weighted imaging (DWI) MRI has been assessed as a modality to evaluate radiation‐induced loss of function in salivary glands in 7 small prospective studies [30, 32, 34, 36, 38, 40]. Dirix et al. evaluated both parotid and submandibular function in a cohort of 8 patients receiving head and neck RT [30]. The mean dose received by ipsilateral and contralateral parotid glands was 53.9 Gy and 20 Gy, respectively. The baseline ADC value (before gustatory stimulation) was higher after RT in ipsilateral parotid glands, and biphasic response after gustatory stimulation was absent. One of the possible explanations for the increase in ADC value is the reduction in acinar (secretory) cells with increased intercellular water [46, 47]. Of note, a negative correlation has been found between SUV and apparent diffusion coefficient (ADC) in studies performing baseline FDG PET‐MRI before head and neck RT [48]. On the contrary, baseline ADC values after RT were not significantly different before and after RT in contralateral parotid glands, suggesting the preservation of the salivary function at doses ranging around 20 Gy. While submandibular glands received in both ipsilateral and contralateral sides a mean dose of 58.5 Gy and 47.5 Gy, no difference was observed between pre‐RT and post‐RT ADC values, at different time points. Shi et al. also demonstrated a significant increase in ADC values in both submandibular and parotid glands after RT, together with a reduction in SEF assessed both at baseline and after gustatory stimulation [38]. Interestingly, a correlation was found between parotid gland ADC 3 months after RT and xerostomia questionnaire (XQ) score. The same findings were also reported by Zhang et al. showing an increase in ADC value after RT in both parotid and submandibular glands [41]. A decrease in ADC was further observed at 1 year in parotid glands, but not in submandibular glands suggesting an absence of salivary function recovery (of note, submandibular glands received a mean dose of 59.8 Gy). Fan et al. also assessed DWI MRI as a follow‐up modality for evaluation of salivary gland function, in patients receiving head and neck RT for nasopharyngeal carcinoma [32]. They demonstrated that post‐RT ADC values were all higher than ADC values pre‐RT. Of note, at all timepoints, ADC in parotid glands was lower than ADC in submandibular glands, probably due to due to their fat rate and the absence of mucous component. Also, ADC value in both parotid and submandibular glands was negatively correlated with SEF. The study led by Loimu et al. was the only one to demonstrate an association between ADC and dose delivered to the parotid gland, with an increase in ADC values 6 months after RT being correlated with doses absorbed by the parotid during RT [36]. However, this study did not confirm the correlation between ADC and SEF, measured with SGS. Juan et al. quantified the evolution of parotid volume and ADC after RT [34]. Parotid glands were significantly smaller after radiotherapy, the most significant decrease being observed at 1.7 months post‐RT (reduction of volume up to 31.2%). Both parotid volume and parotid ADC were correlated with post‐RT xerostomia. Zhao et al. also studied the association between ADC parameters and xerostomia following RT for nasopharynx cancer [49]. They showed that ADC was increased in both parotid glands and submandibular glands at 3 months following RT, and then showed a decrease. ADC remained lower in sublingual and submandibular glands among patients where effort was made to spare the submandibular glands, suggesting that protecting the submandibular glands might have a positive impact on the functional retention of the parotid and sublingual glands. Submandibular protection resulted also in an alleviation of xerostomia symptoms.

A correlation between perfusion parameters and both SGS and clinical outcomes has been investigated within 4 studies. Juan et al. prospectively assessed the alteration of parotid perfusion using dynamic contrast‐enhanced (DCE) MRI [33]. Several perfusion parameters were shown to be modified after head and neck RT (lower Kel and K21, higher peak‐enhancement values, and time‐to‐peak) and correlated with xerostomia severity, suggesting both decreased vascular permeability and increased extracellular and extravascular space in radiation‐induced parotid damage [25]. Of note, the time interval between RT and MRI evaluation was largely variable between individuals, which may hamper the reproducibility of the results of the study. Wu et al. also assessed salivary gland changes after RT, with both ultrasound and DCE MRI [44]. A mean reduction of 25% and 21% in parotid and submandibular glands volume was respectively noted, which was maximal at 6 months after RT. A modification of hemodynamic parameters was also noted with both a decrease in resistive and pulsatility index and an increase in peak systolic velocity and end diastolic velocity, suggesting increased extracellular and extravascular space. There was no correlation between the parotid volume and hemodynamic changes and doses received by the gland. Zhou et al. confirmed a modification in perfusion parameters (perfusion fraction, maximum relative enhancement) within PG after RT for nasopharyngeal carcinoma, reflecting radiation‐induced changes in parotid microstructure [43].

Only 6 studies were led to determine predictive imaging factors for xerostomia after head and neck RT. Marzi et al. performed both anatomical and functional MRI in a prospective study of 89 patients receiving head and neck RT, and correlated imaging parameters to self‐assessed xerostomia questionnaires [37]. Thirty‐three percent of patients experienced grade 2 xerostomia at 12 months. In these patients, the dose absorbed by submandibular glands was 64.2 Gy. The most accurate predictive model for late severe xerostomia was based on cumulated V65 Gy on both parotid and submandibular glands and the integral of xerostomia scores performed from baseline to the middle of RT. Van Dijk et al. also assessed MRI biomarkers and their association with xerostomia 12 months after head and neck RT [22]. MRI texture parameters assessing parotid gland fat at baseline were predictive of late severe xerostomia, suggesting that this imaging modality could help discriminate which patients exhibit parotid radiosensitivity. Zhou et al. assessed parotid damage during head and neck RT through the use of DWI MRI, and showed an early increase in parotid ADC at 5 weeks of RT, while both parotid volume and diffusion kurtosis coefficient declined [42]. A correlation was found between the corrected diffusion coefficient after RT and mean radiation dose, but not with xerostomia severity. Sheikh et al. used both radiomics and DVH values to predict acute radiation‐induced xerostomia [45]. The best model included both CT and MRI parameters, DVH values, and clinical data, with an AUC of 0.79. Zhang et al. showed that salivary glands (parotid and submandibular) showed both an early decrease in ADC values at rest and a lower response to gustatory stimulation at 2 weeks [40]. The modification in ADC values after gustatory stimulation was predictive of the degree of xerostomia at 6 months after treatment, raising the question of the benefit of adaptive RT in this specific population of patients. Shen et al. demonstrated that intravoxel incoherent motion (IVIM) MRI had the ability to predict during RT the risk of radiation‐induced xerostomia [31]. As a result, one can imagine that patients showing early radio‐induced lesions to the parotid glands could benefit from adaptive RT, with a new plan of treatment aiming to mitigate more strictly the dose delivered to the salivary glands.

## 4 Discussion

**FIGURE 2:**
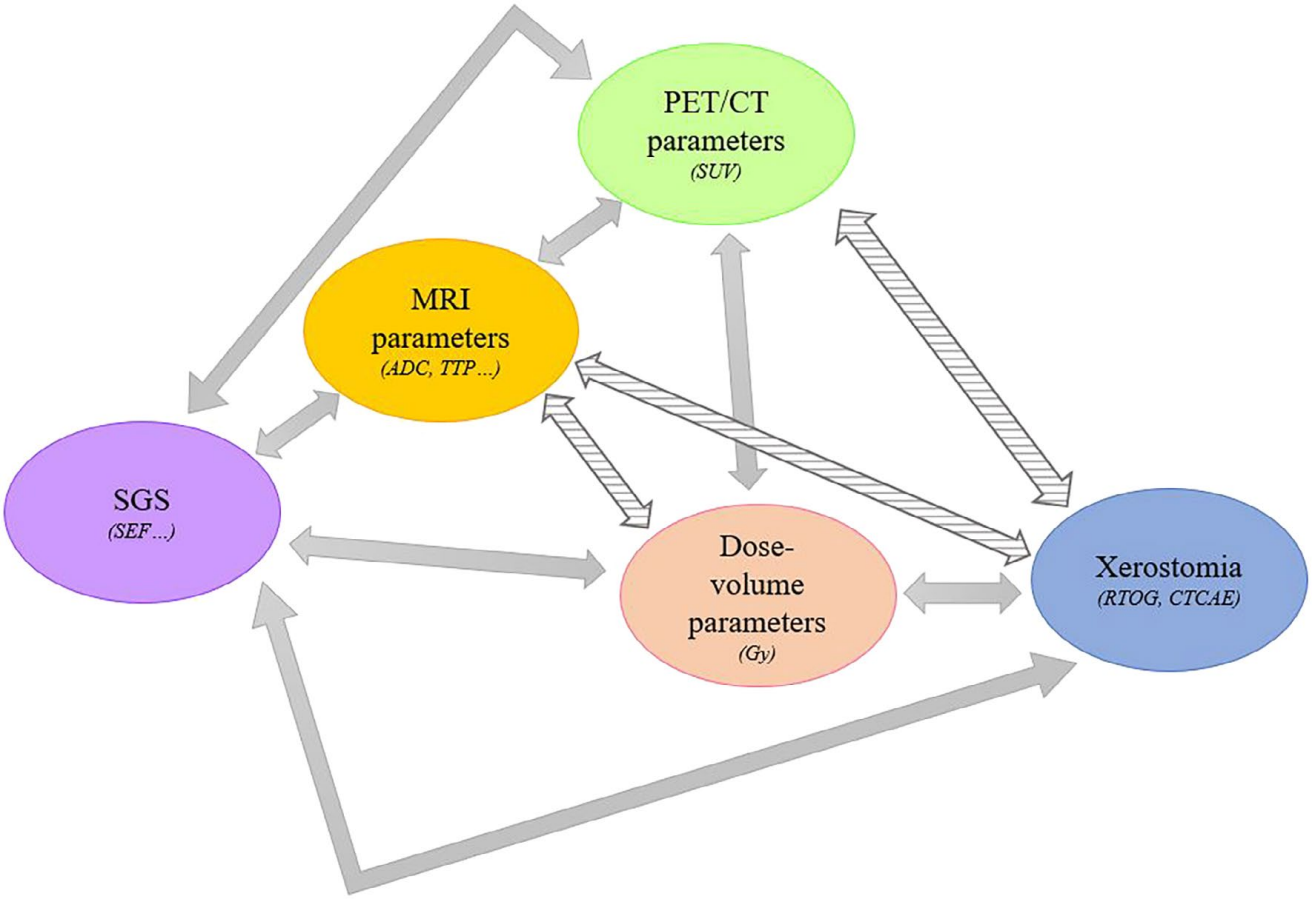
Correlation between the different imaging modalities and dose‐volume parameters (mean dose, volume receiving XGy, etc.)/onset of xerostomia. Full line: Existence of a correlation, dotted line: Doubtful correlation. ADC = apparent diffusion coefficient, Gy = gray, PET/CT = positron emission tomography/computed tomography, SEF = salivary excretion function, SGS = salivary gland scintigraphy, SUV = standardized uptake value, TTP = time to peak.

Our systematic review showed that SEF following head and neck RT is correlated with the dose delivered to both parotid and submandibular glands. Salivary gland SUV following RT has been shown to be correlated with SEF, in several studies. Parotid ADC increases and parotid volume decreases after head and neck RT, and the impact of RT seems to be dose‐dependent [30]. The correlation between ADC and SEF is variable between studies [36]. Perfusion parameters suggest an alteration in parotid microstructure with both decreased vascular permeability and increased extracellular and extravascular space following head and neck RT, but evidence of a correlation between dose‐volume parameters and SEF remains scarce. Models including early SUV decline or ADC increase during RT and clinical parameters can help predict the loss of salivary function after RT. Last but not least, discrepancies exist between studies with regard to the correlation between PET/CT and MRI parameters and the onset of xerostomia (Figure 2). It should be kept in mind that xerostomia is a quality‐of‐life issue, for which the perception might not be similar for all patients.

The management of xerostomia is currently based on symptomatic treatment with artificial saliva, with no existing treatment performed with curative intent. Therefore, the prevention of xerostomia represents a crucial endpoint when planning head and neck RT. Several studies demonstrated the possibility of early detection of salivary gland damage during RT [21, 22, 23, 25, 27, 37, 40, 42, 45], raising the possibility of adapting the treatment plan during RT. Adaptive radiotherapy has demonstrated its ability to reduce the mean dose delivered to parotid glands and therefore represents an innovative approach to minimize normal tissue toxicity [50]. While the ARTIX trial failed to demonstrate the benefit of adaptive head and neck radiotherapy in decreasing the rates of severe xerostomia in an unselected population [51], a subset of patients demonstrating early damage to the salivary glands might still benefit from this approach. Also, a large proportion of patients have been demonstrated to experience xerostomia at baseline, with this proportion rising up to 41% in the study led by Van Dijk et al. [27]. As tailored approaches involving treatment replanification are resource‐consuming, future studies should consider the selection of a population with both satisfactory salivary function and early predictive factors of late xerostomia. Aside from adaptive RT, proton therapy could represent another option to improve salivary gland‐sparing [52]. Indeed, due to its dose distribution characteristics with a sharp fall‐off dose beyond the target volume, it could enable to decrease in the volume of normal tissue receiving intermediate doses and thereby decrease the rate of severe late toxicities. Several retrospective studies showed a low rate of xerostomia and even a reduction in rates of severe xerostomia compared with photon therapy [53, 54]. Prospective trials are eagerly awaited in this clinical setting.

Several innovative salivary gland‐sparing options have been investigated in the past few years. While clinical data have long suggested the ability of salivary glands to partly regain their function after RT, preclinical data showed that salivary glands contain stem cells that are capable of regenerating salivary tissues [55]. Following evidence that the dose delivered to the parotid volume containing major ducts was predictive of late xerostomia in animal models [56, 57], van Luijk et al. confirmed the hypothesis that dose delivered to regions containing stem cells was predictive of xerostomia 1 year following head and neck RT [58]. Parotid gland stem cell‐sparing has been evaluated within a randomized controlled trial, and showed contralateral stem cell region to be highly predictive of grade ≥ 2 xerostomia 1 year after RT [59]. Yet, no significantly better salivary gland function was demonstrated within the stem‐cell‐sparing arm, compared to standard treatment planning. Sample et al. showed that PET/CT radiomic features were able to predict relative importance within subregions of parotid glands, with a low uptake of PSMA being associated with a high dose sensitivity [60]. Interestingly, the data from this study suggests the possibility to perform adaptive treatment planning based on PSMA uptake, which could ultimately lead to a reduction in xerostomia following head and neck radiotherapy. Also, new possibilities for salivary gland‐sparing have been unveiled after the discovery of tubarial salivary glands in the nasopharynx using PSMA PET/CT [61]. Valstar et al. recently demonstrated that the mean dose delivered to these glands was associated with the onset of severe xerostomia at 1 and 2 year following head and neck RT [62]. While data are to date lacking as to both sparing possibilities and constraints to be applied to tubarial glands, their delineation should be considered for dose‐reporting purposes within prospective trials.

Several limitations exist with regard to the conclusions drawn by this systematic review. First, xerostomia has been assessed at different timelines between studies (from 1 month to 2 years after RT), which limits the comparability between studies. Second, salivary function has been shown to recover up to 1 year after radiotherapy [63, 64, 65], yet some of the studies included in this review have a follow‐up of 3–6 months. Also, heterogeneity exists in the PRO used to assess xerostomia (Likert scale of the EORTC QLQ HN35, EAT‐10 questionnaire, XeQOL questionnaire, XQL score, etc.), thereby hampering the comparability between studies. Last but not least, discrepancies exist with regard to the assessment of imaging parameters between studies. For example, the assessment of an ADC value is highly dependent on the b value chosen for DWI sequences, and has been shown to vary between studies. Also, interobserver variability can exist when determining the region of interest (ROI), for both PET/CT and MRI quantitative analysis. Last but not least, the search has been performed only within the PubMed database, and not within other databases.

## 5 Conclusion

SEF following head and neck RT are correlated with the dose delivered to both parotid and submandibular glands. A correlation was demonstrated between SEF and salivary gland SUV following RT. Scarce evidence exists with regard to a correlation between SEF and both ADC and perfusion MRI parameters. Models including functional parameters extracted from either PET/CT or MRI have the potential to predict the onset of late xerostomia, and therefore unveil new possibilities for adaptive treatment in a selected population of patients. Further salivary gland‐sparing trials should however consider restricting these procedures to patients with satisfactory salivary function at baseline.

## Author Contributions

**J. Le Guevelou:** conceptualization; data curation; formal analysis; methodology; project administration; writing – original draft; and writing – review and editing. **X. Palard‐Novello:** conceptualization; writing – review and editing. **E. Kammerer:** writing – review and editing. **M. Baty:** writing – review and editing. **M. Perazzi:** writing – review and editing. **A. Larnaudie:** writing – review and editing. **R. De Crevoisier:** writing – review and editing. **J. Castelli:** conceptualization, writing – review and editing.

## Consent

The authors have nothing to report.

## Conflicts of Interest

The authors declare no conflicts of interest.

## Permission to Reproduce Material From Other Sources

The authors have nothing to report.

## Data Availability

Data sharing not applicable to this article as no datasets were generated or analysed during the current study.
